# Understanding online behavior towards community water user participation: A perspective of a developing country

**DOI:** 10.1371/journal.pone.0270137

**Published:** 2022-07-28

**Authors:** Narongsak Sukma, Adisorn Leelasantitham

**Affiliations:** Technology of Information System Management Division, Faculty of Engineering, Mahidol University, Nakhonpathom, Thailand; Szechenyi Istvan University: Szechenyi Istvan Egyetem, HUNGARY

## Abstract

The social network is a network of virtual relationships that can facilitate the development of a new society in which everyone can use online communication effectively. This article investigates and identifies the fundamental influences on the social network system, as well as the online behavior of the community users. This study was designed by any social network to help improve efficiency and offer people with services that match the needs of their communities. Furthermore, it increases participation in the equitable distribution of social benefits. This study investigates the critical factors that impact a community’s view of community water user participation. The researcher sent a questionnaire on a five-point Likert scale to 1,000 community water customers and collected 627 valid replies. Data from 14 villages were sampled using a simple random sampling strategy to acquire the data. Subsequently, descriptive statistics are used to describe the data (frequency distributions, percentages, averages, medians, and standard deviation). Furthermore, PLS-SEM was used to examine the relationships between factors and to launch the conceptual model using PLS route modeling. This study reveals that digital technologies are crucial to increasing the expectations and happiness of the community through social networks. Multiple causes contribute to its expansion. In addition, this research provides an outstanding case study technique based on TAM and ECT to assess people’s social networking and community participation habits. Additionally, community water providers participate in social networks by certifying that their expectations are met.

## Introduction

Today, everyone is aware of which "social network" influences nearly every area of people’s lives. It is a service similar to a virtual social network that connects many individuals via the Internet, such as Facebook, Twitter, and Blogger. Moreover, social networking is currently a prominent marketing tool and an online community [1]. A social network is a tool that facilitates communication and saves people time by facilitating the transmission of information. It is also a trading channel that entrepreneurs use today to create income through the sale of goods and services [2]. In addition, the social network has improved social contributions by building numerous online communities to assist one another, allowing individuals to share their experiences and information, and in particular, by forming an online community of experts [3]. Bring each professional field together to answer advice-seeking individuals’ questions and assist each other. The online community is a virtual community that allows everyone to share their ideas and upload information to separate forums. In addition, the Internet fosters social change in a variety of domains [4]. A fundamental advantage of social networking is the power of public administration [5, 6], which is employed in monitoring and as a conduit to express thoughts on numerous situations in which the public has reservations about the work of organization officials. Several organization departments were found to have used social networking to improve their communication channels and improve the transparency of their public jobs [7]. A social network is a tool that improves workplace transparency, which is a vital step in combating corruption within a company. It helps organization employees be accountable and conduct organization operations effectively and efficiently so that everyone is treated fairly[8].

Therefore, organizational offices must have strategies and guidelines for establishing concrete transparency to obtain respect and confidence [6]. The organizational unit responsible for the supply of water to the people is regarded as a crucial organizational entity as it is responsible for the essentials of the people, namely the water that people consume daily. Every residence must cope with a water supply issue, which might vary based on the circumstances [9], such as water that does not flow, smells terrible, or contains silt, which can also be a problem for various reasons [10]. However, there is only one route for citizens to contact the organization official responsible for registering complaints and reporting documents on the official day and time. Moreover, the worst aspect is that citizens are unable to monitor the processes of government officials after receiving complaints. Alternatively, suppose that there is an online community of users leading people in each village. For this study’s purpose, all parties can use the online community to communicate between water consumers and organization officials. The online community is intended to record the presence of irregularities in the water supply and as a means of alerting organizational entities, such as informing them in advance of the maintenance schedule of the water system. As a result of establishing an online community for community water supply, people and organizations’ offices should be better able to collaborate and encourage work transparency that can be better monitored. By the way, this study focuses mainly on transparency issues that will have an effect on the economy of the community and encourage participation. In addition, increased involvement has been found to help resolve ethical dilemmas through the use of good governance and transparency technologies available through social networks today. Numerous articles call for the convergence of good governance and business management to create trust and deliver solutions. Additionally, [11] describes social networks as a form of governance as the process of selecting, holding accountable and removing organization officials; the safeguarding of individual rights; and the organization’s ability to design and implement policies. Effective organization occurs when it addresses the collective concerns of its constituents and meets their needs. Participation is a necessary requirement for successful governance. Salminen highlights the importance of fully implementing good governance for the common good. Existing research has examined user participation in online communities using a variety of theoretical frameworks, including the Technology Acceptance Model (TAM) and expectation confirmation theory (ECT). The authors discover that user behavior is significantly influenced by perceived usefulness, trust, and self-efficacy. However, little attention has been paid to the aggregate influence of an online community on user behavior. A virtual community is made up of people who share common interests. [12] explains why stakeholders are a critical success factor in environmental management. They communicate with each other to resolve issues, exchange ideas, and seek help. Thus, an individual member’s conduct might be influenced not just by other water users’ incentives, such as perceived utility, but also by the behavior of other members and the community.

By the way, no previous study has investigated every significant feature of a single workplace. They made no attempt to determine the relationship between social networks, transparency, stakeholders, and community value creation in the community water industry. Also, the goal of this study is to investigate online community water user engagement through the lenses of TAM and ECT. In particular, this study found a relationship between three factors that had not been known before: social networks, transparency, and stakeholders. All three of these factors help water users join the online community, which increases the value of local goods and services by a large amount. As a result, the following research question:

RQ1: How can social networking enhance water user participation in the community?
RQ2: How does online community participation intention influence the satisfaction of e-services?

In order to provide a response to the research question, we combed through the previously published research and the theoretical background of this study. After that, the research model and hypotheses for the proposed study are outlined. Following this, we conduct an experiment to verify the validity of the proposed model by using it to analyze a large dataset that we have assembled. Lastly, we look at how the results of our study contribute to the development of theories and how we can learn more about how users continue to participate in activities hosted by online communities through user engagement.

## Theoretical discussion

### Community water supply business

A literature review of the community water supply business community water is determined. To begin with, public utilities operate under a more traditional management model, with the organization building and managing all communities. It is a style of bureaucratic management defined by a highly complicated organizational structure. Additionally, [13] explains that central organization policies were incompatible with the context of each location, resulting in the community water supply system’s being unable to address the community’s desired issues. As mentioned by [14], a management issue comprises the exercise of authority and responsibilities in order to benefit specific groups of people in order to obtain preferential access to water. Second, privatized utilities are those that are subsidized by the organization to be managed by private companies. The benefit is that the organization is highly adaptable and self-managing. On the other hand, [15] discovered problems with pricing transparency and a lack of checks and balances, resulting in people not having equitable access to water and selling at prices we could not verify. If this is the case, private companies may be allowed to establish water delivery prices without recourse to the public sector for a better deal. Third, PPPs (public-private partnerships) are a modern management paradigm that is well-suited to large-scale infrastructure and public service projects across a variety of industries. Operational costs are substantial and may be insufficient if fully funded by the public sector. One of these types of joint ventures, however, has a number of drawbacks, one of which is governance. Formal investment projects are more challenging to organize than negotiating with state-owned firms. Without a properly written and implemented policy, the PPP will fail and become ineffective. This is because risk must be shared between the public and private sectors [16, 17].

The purpose of this study was to identify strategies to close some of the gaps in the current community water supply business by increasing online community water user participation, with a particular emphasis on the extent to which water customers can participate via online communities. Because social networks have become more accessible, the way online communities encourage users to participate has changed.

### Technology Acceptance Model (TAM)

TAM is a behavioral model that was first established to understand computer usage [18]. Because of its resilient features, TAM is the most often used model to characterize an individual’s adoption of a particular information system. [19] presented the notion, which has since been used in an avalanche of research to predict and explain why consumers accept or reject information technology [20]. To this end, TAM introduced two new factors: perceived utility (PU) and perceived ease of use (PEOU), which were significant predictors of attitudes toward certain technologies. [19] suggested that new external factors be constructed to account for perceived usefulness and perceived ease of use. The components PEOU and PU, identified by [21] as the most relevant in explaining the use of the system, are the most significant in the model. Although it is widely used, some researchers have suggested that it is necessary to include additional variables to build a more robust model. [22] proposed to remove attitudes against a particular technology from their planned expansion due to the low predictive capacity of attitudes toward that technology for behavior of behavioral intention and actual use of the system. Many further investigations have supported and substantiated the findings. Several research investigations have been carried out on this subject [23]. Since a broad group of people first used it, TAM has been used in many technologies, in many locations, and with a wide range of different control components. It has a well-deserved reputation for being reliable. Additionally, the authors pointed out that the TAM is a comprehensive mediating model that primarily accounts for the effect of PEOU on behavioral intention via the use of probability.

Another In a meta-analysis of 145 published research publications [24], the authors found that three key factors contribute to the popularity of TAM. The model is specifically designed for information technology and is an excellent tool for understanding and predicting the adoption of a wide range of technologies by a large user population across various organizations, cultural contexts, and levels of expertise. Second, TAM has a well-established theoretical framework, significant research, and an inventory of measuring scales that have been independently confirmed. Third, because of prolonged research, the model has collected a large amount of empirical data to support its explanatory powers and characteristics. Consequently, it is often the prevailing paradigm for user acceptance of technological innovations. The existing study on TAM should be argued with the ECT and the new principal constructs in this study.

### Expectation Confirmation Theory (ECT)

In their systematic literature review, [25] established the ECT theory to investigate post-purchase behaviors such as repeat purchases, complaints, and service marketing. It is often used in market research to assert that customers will form expectations before purchasing a product and will develop knowledge of the product’s real effectiveness after usage. If the expectation is fulfilled, the condition is satisfied. If an expectation is not fulfilled, it will remain unfilled. ECT theory is often utilized to extract meaning and create predictions. As shown in [26], consumers are satisfied and want to buy again. Repeat purchase intent is determined by past satisfaction. Consumer satisfaction is considered critical to establishing and maintaining long-term customer loyalty. This idea was very predictable. [27] shown by the fact that people repurchase a variety of different goods and services. These include the purchase of a multi-cycle automobile, the acquisition of a video camera [28], and the management of the restaurant for future usage by customers [29]. Additionally, it refers to expectations as a factor in customer satisfaction choices. This is because service expectations are used to determine how long customers will consider a product or service before re-purchasing it [30]. ECT outlines a product’s or service’s repurchase expectation and its confirmation or denial depending on the product’s or service’s performance, which may affect customer satisfaction (Islam, 2014). ECT has been used in a variety of products and sectors, including online banking information systems and restaurants. Consumers have expectations about items or services, and then their actual use of these products or services enables them to validate or refute these expectations [31].

According to the review of pertinent literature, which indicates that ECT has never been researched in a community water system, this study is an extension of ECT to understand user participation in the community water system. It should be disputed with the TAM. If the product’s or service’s actual performance exceeds the expectation, the expectation is verified. Similar to [32], consumer pleasure and post-purchase behavior depend on their expectations being confirmed or disproven. On the other hand, the actual performance falls short of expectations, and the expectation is invalidated.

### Research model and hypotheses

**Fig 1:**
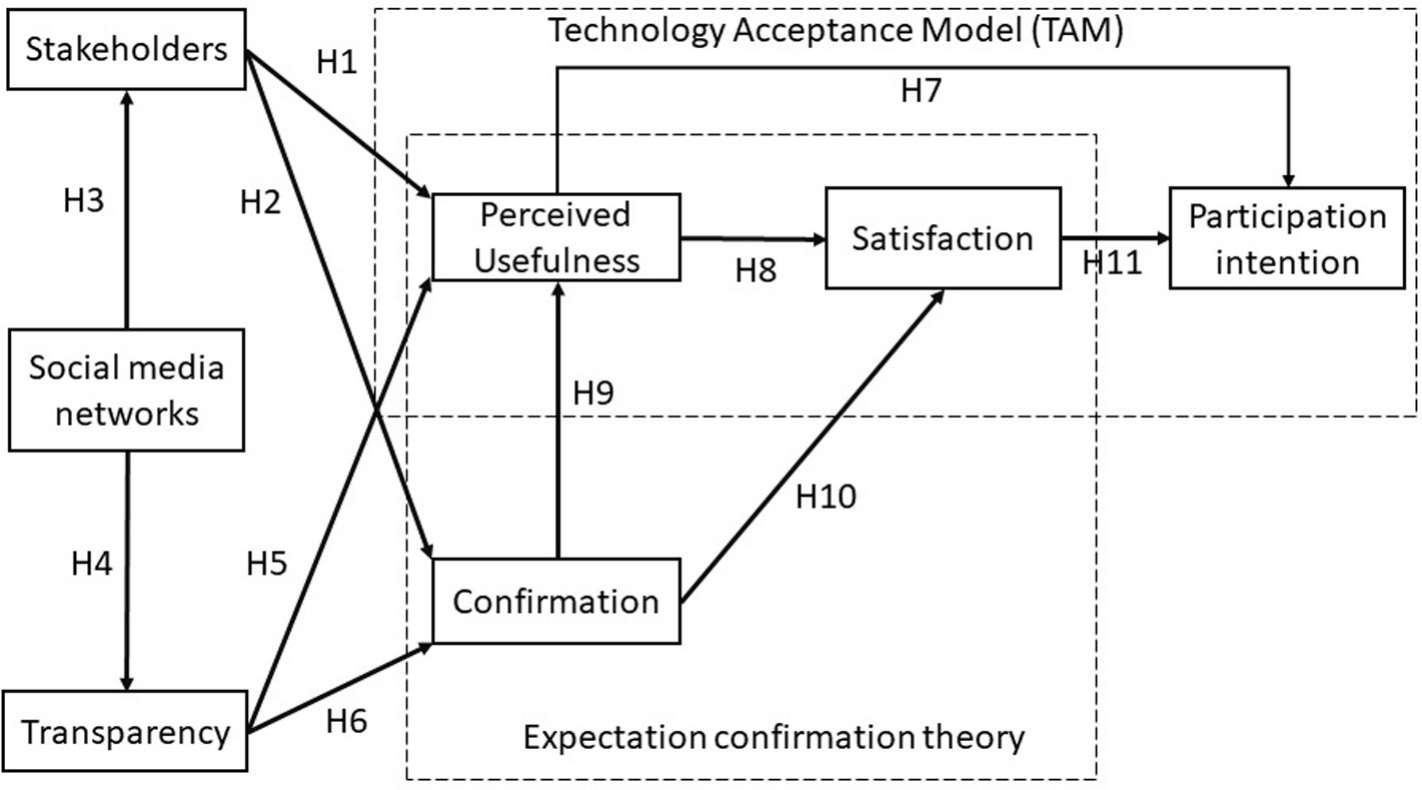
The research models.

As discussed previously, the existing community water supply industry has encountered several administrative and management obstacles. To begin with, all organized public, private, and public-private partnerships have advantages and disadvantages, offer limited options, and preclude independent businesses from competing. Second, the research model designed to look at the participation of water users in online communities through the lens of TAM and ECT includes three constructs (social media, transparency, and stakeholders) that reflect satisfaction and participation intention constructs. (See Fig 1).

This study will test 11 hypotheses to address the challenges listed in the research question by analyzing three aspects derived from the review of the literature: stakeholders, transparency, and social networks using an integrated model including TAM and ECT as the two main theories. Consequently, we expect to create a model for the existing social online platform that would provide superior service and quicker replies. Through a well-managed and controlled trust information management system, this model must be extremely dependable and capable of being audited for transparency by relevant parties.

### Stakeholders

According to stakeholders or participants, the impact of an actor within a network is contingent on the dependency of other players on the resources they control [33]. Moreover, according to the IS participation hypothesis, stakeholders have a role in the success of an information system. Participants are subgroups of stakeholders asked to participate in the solution’s conception and/or execution. Participants and stakeholders may differ in many ways. Furthermore, [34], it may be difficult to choose suitable participants from the group’s composition. There may be a correlation between the success of solution creation and the execution of solution. In addition, stakeholders are critical success factors because they provide considerable new information and insights about the circumstances, limitations, and possibilities of stakeholders. Consequently, we have the following hypotheses:

ST:
  ◦ H1, Stakeholders positively perceived the usefulness
  ◦ H2, Stakeholders positively influence confirmation

### Social network

A social network is a collection of connections between people, objects, and events. Due to the diversity of relationship types, various networks may be constructed using the same building blocks [35]. When a business creates an information technology system, network centralization is a crucial element. Individuals in the network center are able to regulate the circulation of resources and related information. Individuals exercise influence over their own authority and resource control within an organization; they are not affected by others. [36] began incorporating social network concepts into an organization’s technological innovation dissemination model. In addition, social exchange theory is used in several studies [37] to explain how social networks operate in return for social benefits and community support. Moreover, strong positive correlations were established between the commitment-trust theory and the Social Exchange Theory with regard to the co-creation of brand value in the social commerce community. Increasingly, businesses are advertising their products and services through social commerce. Therefore, social networks are considered relevant by the study. Consequently, we develop the following hypotheses:

SO:
  ◦ H3, Social Network positively influences stakeholders
  ◦ H4, Social Network positively influences transparency

### Transparency

According to a review of the relevant literature, the management of the water supply business has a significant challenge in the area of ethics, with social and legal concerns deeply related to ethical issues. To solve this issue [38], we conducted research and found several articles that suggested integrating transparency, good governance, and social networks into the management process would help address ethical challenges and improving trust [39]. According to some intriguing studies [40], followers’ perceptions of confidence in a leader as well as followers’ judgments of the leader’s performance improved when the leader displayed optimism and transparency. Several positive correlations between transparency and trust. This may be done to foster confidence in a leader or organization and to demonstrate that an ethical problem such as this may be handled well by excellent administration. As a result, the importance of maintaining open communication is emphasized throughout the investigation. The following are some assumptions that we have developed as a result:

TP:
  ◦ H5, Transparency positively influences perceived usefulness
  ◦ H6, Transparency positively influences confirmation

### Perceived usefulness

Perceived Usefulness is part of ECT and has been used in a variety of research initiatives, including those that included online commerce and continued intention on the Web [41]. In the course of [42] research into the user’s ongoing intentions in relation to the online site, the researcher also made use of playfulness to broaden her experimental design (ECT). Playfulness, contentment, a user’s perception of how helpful a website is, and customer satisfaction are all characteristics that play a significant role in determining whether or not a user intends to return to a certain website. According to the findings of another study [43], the most common way for patients to learn about ECT is through referrals from patients. The results indicated that the user’s desire to continue using a product had a positive influence on both the user’s perception of the product’s usefulness and the user’s pleasure in using the product. The four basic structures of ECT are the expectation confirmation construct, the perceived utility construct, the satisfaction construct, and the continuing intention construct. Confirmation is defined as the degree to which a user believes that their original expectations are being confirmed in the way in which they were first communicated. Confirmation occurs during real usage of the product. As a consequence of this, it is important to clarify, in the context of post-adoption, that users’ expectations develop as a direct result of their experience and that post-adoption expectations will be based on the actual use experience of the users. Perceived usefulness of something is a second important component of the notion of expectation confirmation. According to, when examining user behavior during both the pre-adoption stage and the post-adoption stage, it is discovered that perceived usefulness is a stable variable. [44] in research conducted in the past, confirmation of expectations has been shown to have a considerable impact on people’s judgments of the utility of products and the level of enjoyment they get from them. As a result, the theories that we have are as follows:

PU:
  - H7 Perceived Usefulness have positive influences participation intention
  - H8 Perceived Usefulness have positive influences satisfaction
  - H9 Perceived Usefulness have positive influences confirmation

### Confirmation

In addition, confirmation has an indirect influence through the commitment of the relationship [45]. The word "commitment to a relationship" (or simply "commitment") refers to an individual’s or organization’s ongoing desire to maintain a business connection that is advantageous to both parties [46]. Dedication to a connection develops when one of the individuals involved in the exchange feels that sustaining the relationship is of the utmost importance and will call for the greatest amount of effort or commitment. However, it is very important to keep in mind that not all social contacts result in the formation of long-lasting, trusted connections. To encourage commitment on the part of the exchange partner, the relationship must be seen as meaningful and significant. To put it another way, for there to be a basis for reciprocity, the resources that are traded must be of some tangible benefit to the party being traded with. Although the sample sizes were small, several studies have been conducted to investigate the topics of commitment and trust in higher education. According to [47], the factors that influence a student’s commitment to their educational establishment are the perceived benefits of attending; the perceived similarity between the school and the students; the trust between faculty and students; and the quality of communication between the students and the educational establishment. This adds to our understanding of the ways in which consumer engagement behavior is influenced by the dynamics of connections and communities. The results will help to better understand how to build service organizations in order to optimize service value and brand loyalty over time. This will be an important outcome of the research. Furthermore, the dynamic between trust and commitment is shifted unexpectedly through social media [48]. As a consequence of this, the hypotheses are stated in the following format:

CO:
  ◦ H10, Commitment positively influences satisfaction

### Satisfaction and participation intention

Satisfaction is the emotional state of a client that is related to a certain service and has an influence on the customer’s motivation. After community water users have had a favorable experience with electronic participation, their motivation to use social networks increases, resulting in their continued use of the community water system. If community water customers are satisfied with the social network based on their previous usage, they will build a high degree of trust and continue to use it [49]. According to [50], users who are satisfied with an information system are more likely to continue using that system. [Citation needed] When a customer is satisfied with a service, they are more likely to be motivated by their own intrinsic interest in the service, its importance, avoidance of guilt, feelings of worth, compliance with rules and avoidance of punishment (intrinsic regulation) and other self-determined motivational factors [51]. This is the most self-determined kind of motivation, and it refers to acts that are carried out with the express aim of delivering delight, interest, and contentment to community water users. Studies done in the past have shown that there is a positive association between satisfaction and intrinsic motivation. A positive association was found between introjected regulation and increased effort expenditure. However, this association was also shown to be related to increased anxiety and worsening of failure management. Identified regulation is a kind of extrinsic motivation that may be characterized as a mainly autonomous regulating style.

Several studies have found participation intention to be discovered and that the popularity of TAM can be attributed to three primary qualities [24]. Subsequently, [20] explained how intensive study has led to the accumulation of a considerable amount of empirical data to support the explanatory power and features. It is common practice to produce projections using models of the adoption of new technologies [21]. This suggests that there are only two aspects that impact the acceptability of the user’s computer system. [Citation needed] The perceived usefulness of the system and the ease with which it may be used are two aspects that play a role in computer adoption. In TAM, the sentiments, and perceptions of its users about the system’s usefulness help to build a favorable attitude toward the system. As was said before, perceived utility (PU) in terms of boosting their own performance was taken into consideration. Additionally, customers have the impression and belief that the system is easy to use. The term "perceived ease of use" refers to the notion that the user does not have to make any effort to understand that the system is still functional. As a result, the theories that we have are as follows:

SA:
  - H11, Satisfaction positively influences participation intention

## Research methodology

### The scope of survey

According to the study on population data for the year 2021, approximately 80% of the people living in the province of Nakhon Phanom made use of the community water supply. Furthermore, the study claimed that the Phanom district had problems with the quality of the water used for household purposes. These problems were especially prevalent in the Na Thon subdistrict, which is not served by the Provincial Waterworks. For the purpose of this study, a simple random sampling strategy was used. The sampling ratio for this study was 14 villages and 2,584 households (every village in the Na Thon subdistrict was sampled), and it comprised all samples from residents of the study region who had resided there for a minimum of one year. This indicates that samples were collected from each of the localities located within the Na Thon Subdistrict (14 villages).

### Questionnaire design

Relevance was analyzed into 7 constructs, and then 21 questions were selected from those structures to be included in the questionnaire. All data subjects gave their consent to data collection and experimentation. The questionnaire, which can be found in the Supplemental Information section, was used to collect data from those who had shown interest in participating. To determine the necessary number of samples, the Taro-Yamane formula was used. It included 2,584 households with an acceptable margin of error of.05. According to estimates, the sample size should have been 347 respondents, which is what was required to collect this research. However, thanks to the incredible participation of communities, we received 627 respondents, which is 180% of the requirement’s sample size. This provided sufficient data to proceed to the next level, where the model and the research hypothesis will be evaluated. The quantitative evidence supports the hypotheses that the research model proposes. The following questions will be included in the following sections: It is necessary to enter information on the individual’s gender, age (in years), level of education, and the amount of time they use the community water supply. A look at the factors that influence the level of community cooperation in prosperous areas. The degree to which communities are able to work together effectively is directly proportional to the degree to which they can do so.

### Data testing and institutional review board: IRB

In addition, after undergoing pilot tests and further modification, the questionnaire was published in 1,000 copies and sent to the mailboxes of all households with even numbers. Participants were instructed to return completed documents to a return box that was placed in a position that was convenient for them, such as the multipurpose building in the village. This process ensured that the questionnaire had enough participants and data reliability for further studies. Furthermore, the sample size of each resident in the study zone was counted and that number was used to calculate the sampling ratio for each community included in the investigation. 627 respondents filled out the questionnaire for this research. All completed questionnaires underwent a thorough inspection to ensure everything was accurate. In the questionnaire document, the researcher describes the conditions for data collection and destruction, of which participants were informed and gave consent to collect data for research. Given these points, consent documentation was provided through questionnaires, and all questions included in the research protocol were revised and approved by the IRB. This study was classified as low risk and exempted from the Mahidol University central institutional review board (MU-CIRB exemption protocol number: 2021/362.1108), which can be found in the Supplemental Information section, because it did not record information that could readily identify the subject (direct or indirect/linked).

### Statistical data analysis

The results of the questionnaire were imported and cleaned up in preparation for the structural equation modeling (SEM) analysis that was to follow. It will be used to perform partial least squares structural equation modeling (PLS-SEM) using SmartPLS 3.3.0 software [52], which will include the measurement model, the fit of the structural model and the model [53]. The last part of the study involves examining descriptive and inferential statistical analysis, which will be examined and discussed in the discussion section. This step follows the collecting, processing, and analysis of questionnaire data, which was the previous step. After that, the data will be easier to understand and more specific. In order to evaluate the study model and questionnaire for their reliability and validity, we are using SmartPLS to analyze some of the least square data sets that we have collected. In particular, this methodology was chosen for this investigation because it is an element-based statistical tool for the creation of causal models and has the potential to be applied to the study issues that are now being considered [54]. PLS is a method of modeling structural equations that saves time and effort by evaluating measurement data and structural models in a single phase of analysis. PLS is also known as the partial least squares method. We decided to use PLS rather than covariance-based SEM techniques such as LISREL because it not only requires a smaller sample size and indicator distribution, but also produces more accurate estimates [55–57]. To investigate the reliability and validity, a method of iterative data analysis consisting of two stages is used. In the first step of the process, the reliability and validity of the measurement model are investigated and analyzed. After that, the structural model is examined to see whether it is capable of representing a hypothetical relationship in as accurate a manner as is practicable.

## Results

In order to assess the reliability and validity of the research model and questionnaire, SmartPLS is being used to select least-square data sets that have been obtained. To be more specific, this technique was selected for this investigation because it is an element-based statistical tool for generating causal models that has the potential to be used for the research questions currently being discussed. PLS is a method for modeling structural equations that examines measurement data and structural models concurrently, thereby reducing time and effort. PLS is also known as partial least squares. PLS was chosen because, in comparison to covariance-based SEM approaches such as LISREL, it needs a lower sample size and indicator distribution while providing a more accurate estimate. In addition, PLS may provide results more quickly. An iterative two-stage data analysis technique is used to assess the reliability and validity of the measurement model. In the last step, an investigation of the validity and reliability of the measurement model is carried out. After that, the structural model is looked at to see if it can show a hypothetical connection with as much accuracy as possible.

### Descriptive analysis

The demographic characteristics of 627 community water users are shown in Table 1, which explains that the percentage of male participants made up 42.9% of the population, while the proportion of female participants made up 57.1 percent of the participants. The majority of the respondents, 60%, were between the ages of 30 and 50 years old. When the levels of education were taken into account, it was discovered that roughly 71% of them had a qualification that was lower than a bachelor’s degree. In conclusion, while taking into account the participants’ prior experience with community water consumption, roughly 61% of the total participants had had prior experience with community water usage for more than 15 years. The information used in this study came from people who had spent their entire life in locations that relied on community water supplies. In order to get an accurate picture of the situation, there was a statistically sufficient gender and age distribution in each of the groups that participated in the study. There is a significant connection between the many elements that play a role in determining the research goals.

**Table 1:** Demographic data of respondents, total (N = 627).

Characteristics	Values	Frequency	Percent (%)
Gender	Male	269	42.9
	Female	358	57.1
Age (years)	18–30	101	16.1
	30–40	182	29.0
	41–50	187	29.8
	> 50	157	25.0
Education	< bachelor	446	71.1
	bachelor	148	23.6
	> bachelor	33	5.3
Community water usages (years)	< 5	38	6.1
	5–10	128	20.4
	11–15	79	12.6
	> 15	382	60.9

### The measurement models

We obtained Cronbach’s Alpha scores of between 0.783 and 0.820, which is above the minimum requirement of 0.7. Using the findings of the internal consistency testing, a composite reliability score (CR) score of 0.874–0.893 was produced, which is acceptable since it is above the threshold of 0.70. The extracted average variance (AVE) should have convergent validity greater than 0.50, while the model yielded AVE values between 0.698 and 0.737 (see Table 2). Additionally, Table 3 includes special information. The median, mean, standard deviation, loading, and variance inflation factor. Also, VIF scores range from 1.523 to 2.163, which is less than 5. The fact that all predictive variables have an association coefficient of less than five explains why they are all suitable [58, 59]. Consequently, none of the variables included in this study to generate predictions was multicollinear. In addition, we evaluated the discriminant validity of the model using [60]. Each diagonal value inside a construct must exceed the sum of the column values by at least 0.70. For example, the square root of AVE for confirmation (CO) is 0.718, which is more than the association with the other constructs, which ranged from 0.698 and 0.737. Thus, the research model satisfies the criteria of a model. The findings generated by the Fornell-Larcker criterion are reported in Table 4. Furthermore, discriminant validity testing as a criterion for assessing associations of latent variables has gained considerable recognition. For establishing discriminant validity, the Fornell-Larcker criterion and cross-loading analysis are two of the most commonly used methods. The ’Heterotrait-Monotrait ratio’ (HTMT) is a second approach to using the multitrait-multimethod matrix, as shown in Table 5. HTMT refers to the sum of all cross-variable correlations among indicators. As recommended by [61], the HTMT should be smaller than 0.85; based on Tables 3–6, all requirements are met, establishing discriminant validity.

**Table 2:** Construct reliability and validity.

Constructs	Item code	Cronbach’s alpha (> 0.70)	Composite Reliability (CR) (> 0.70)	AVE (> 0.50)
Confirmation	CO	0.804	0.884	0.718
Participation intention	PI	0.787	0.876	0.701
Perceived Usefulness	PU	0.797	0.879	0.709
Satisfaction	SA	0.820	0.893	0.737
Social networks	SO	0.791	0.877	0.705
Stakeholders	ST	0.783	0.874	0.698
Transparency	TR	0.816	0.891	0.731

**Table 3:** The reliability and validity of the results.

Construct		Question items	Mean	S.D.	Loading (>0.70)	VIF (<5.00)	Adapted from
Perceived Usefulness	PU01	You think that social networks help you do your job faster and make it easier to find the information you need.	3.805	0.828	0.796	1.643	[62–64]
	PU02	You think that social networks help you to complete activities more rapidly.	3.949	0.750	0.879	1.774	
	PU03	You believe that social network is beneficial and can help you perform better at work.	3.931	0.779	0.848	1.686	
Satisfaction	SA01	You are satisfied with your social network experience.	4.104	0.809	0.883	2.163	[30, 65]
	SA02	you satisfied with your capacity to utilize social network features	4.131	0.827	0.879	2.112	
	SA03	You think you made the right decision to use social networks.	3.994	0.795	0.810	1.566	
Confirmation	CO01	You are perpetually willing to help with community events.	4.041	0.817	0.855	1.788	[66–68]
	CO02	You take pride in your community membership.	4.016	0.808	0.845	1.803	
	CO03	You believe it is critical to establish and maintain positive relationships with community-based organizations.	4.037	0.764	0.841	1.634	
Social networks	SO01	You are always on social networks.	3.841	0.822	0.848	1.670	[69, 70]
	SO02	You use social media to stay informed and connect with loved ones.	4.094	0.716	0.855	1.733	
	SO03	You think that social media makes life easier.	4.155	0.626	0.815	1.607	
Stakeholders	ST01	You think bringing people together for activities helps the community’s image.	4.019	0.723	0.865	1.834	[71–73]
	ST02	You think that local and community activities help the community to work together and improve its economic well-being.	3.939	0.766	0.819	1.575	
	ST03	You think it’s vital to share responsibility for community and environment.	4.064	0.794	0.821	1.583	
Transparency	TP01	The community should know and understand the rules before they are implemented, you believe.	4.062	0.834	0.868	1.852	[74, 75]
	TP02	You think everyone should consider public topics like income and expenses.	4.121	0.854	0.861	1.963	
	TP03	You think the organization office should share accurate data in an easy-to-read format.	4.072	0.816	0.835	1.679	
Participation intention	PI01	You intend to continue utilizing social network rather than personally visiting an organization office.	4.027	0.754	0.863	1.752	[30, 63]
	PI02	You plan to continue utilizing social network rather than discontinue doing so.	4.041	0.800	0.855	1.738	
	PI03	You want to continue to use social network in the future	3.876	0.745	0.793	1.523	

**Table 4:** Fornell-Larcker criterion.

Constructs	CO	PI	PU	SA	SO	ST	TP
Confirmation (CO)	0.847						
Participation intention (PI)	0.535	0.838					
Perceived Usefulness (PU)	0.466	0.401	0.842				
Satisfaction (SA)	0.636	0.601	0.480	0.858			
Social networks (SO)	0.544	0.466	0.441	0.512	0.840		
Stakeholders (ST)	0.627	0.569	0.465	0.674	0.517	0.835	
Transparency (TR)	0.599	0.522	0.504	0.658	0.493	0.626	0.855

**Table 5:** Heterotrait-Monotrait ratio (HTMT).

Constructs	CO	PI	PU	SA	SO	ST	TP
Confirmation							
Participation intention	0.671						
Perceived Usefulness	0.566	0.497					
Satisfaction	0.783	0.744	0.578				
Social networks	0.677	0.587	0.550	0.637			
Stakeholders	0.787	0.724	0.582	0.841	0.657		
Transparency	0.738	0.646	0.612	0.803	0.609	0.781	

**Table 6:** Results of the structural model.

Hypothesis	Path	Path coefficient (β (>0.10)	t-value (>1.96)	p-value (<0.05)	Inner VIF (<5)	Decision
H1	Confirmation -> Perceived Usefulness	0.190	3.711	0.000	1.863	Supported
H2	Confirmation -> Satisfaction	0.527	15.076	0.000	1.277	Supported
H3	Perceived Usefulness -> Participation intention	0.146	3.948	0.000	1.299	Supported
H4	Perceived Usefulness -> Satisfaction	0.234	6.657	0.000	1.277	Supported
H5	Satisfaction -> Participation intention	0.531	13.919	0.000	1.299	Supported
H6	Social network -> Stakeholders	0.517	14.004	0.000	1.000	Supported
H7	Social network -> Transparency	0.493	13.000	0.000	1.000	Supported
H8	Stakeholders -> Confirmation	0.414	10.414	0.000	1.644	Supported
H9	Stakeholders -> Perceived Usefulness	0.168	3.616	0.000	1.962	Supported
H10	Transparency -> Confirmation	0.340	8.936	0.000	1.644	Supported
H11	Transparency -> Perceived Usefulness	0.286	5.497	0.000	1.859	Supported

### Structural model

**Fig 2:**
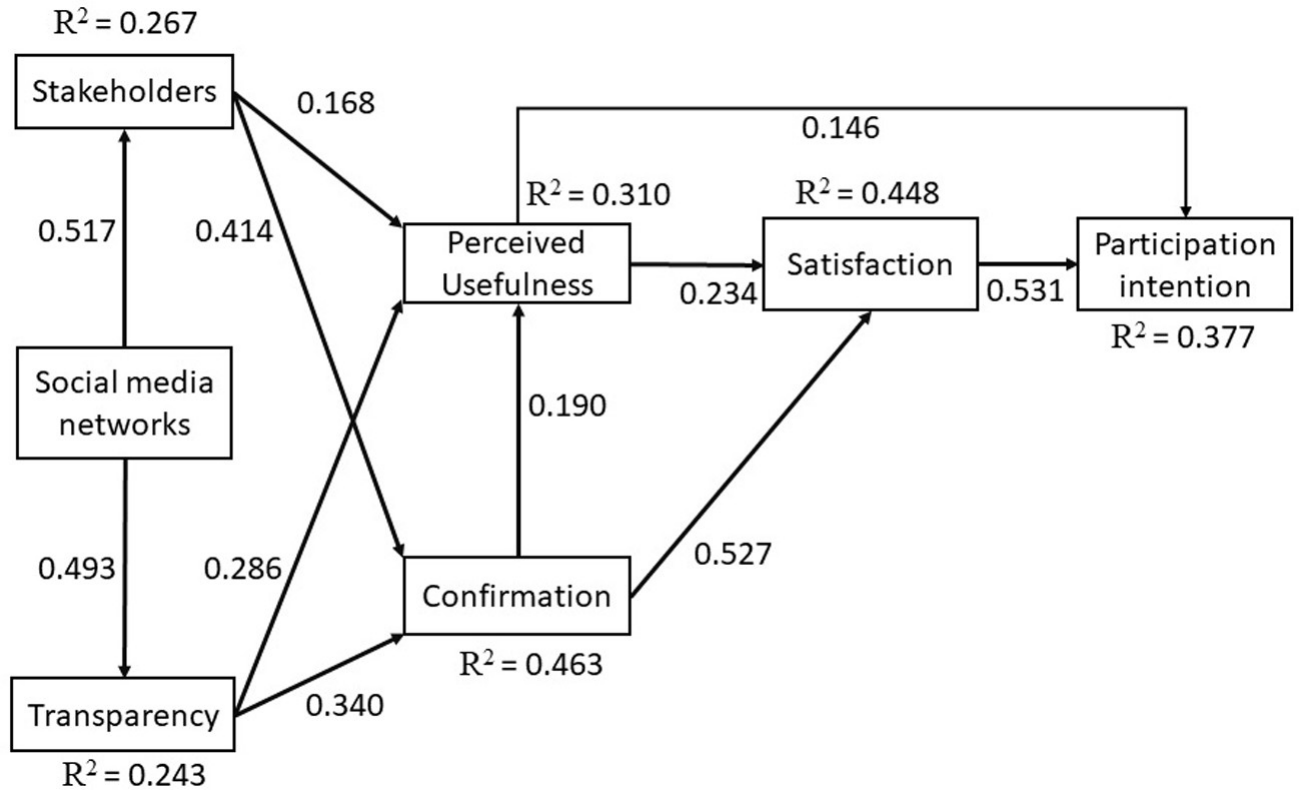
PLS algorithm results.

After receiving findings that were satisfactory from the earlier assessment, we used SmartPLS 3.3.0 to test the hypothesis and determine the degree to which the data were goodness of fit (GoF). In this part, we will examine and evaluate the assumptions that form the basis of the recommended research model that was covered in the research model section. We used a bootstrapping method with 5,000 resamples and a significance threshold of 0.05 for the path coefficient, the t-value and the p-value. This method was recommended by [56].The path coefficient must be greater than or equal to 0.10, the t-value must be greater than or equal to 1.96, and the p-value must be less than or equal to 0.05 to meet the acceptance criteria [55–57]. As a consequence of this, the data suggests that the remaining hypotheses are H1, H2, H3, H4, H5, H6, H7, H8, H9, H10, and H11, which have all been validated. Table 6 provides a summary of the results, and Fig 2 provides a visual representation of the output of the model together with an indication of the hypothesis testing performed by the SmartPLS program. In terms of how well the model fits the data, we got a score of 0.501 for goodness of fit.

### Model fit

The results of each construct that was included in the proposed model have been reported, as was covered in the structural model section. In this section, the results of the structural model that was built using SmartPLS have been analyzed, and data has been taken from all of the different structures of the model that has been presented (see Fig 2). Table 6 contains the findings of the structural model that was created using SmartPLS. The model fit of an empirical investigation is made up of the following three components: To begin with, the coefficient of determination, or R^2^, is considered undesirable when it is less than 0.19, considered bad when it is between 0.19 and 0.33, considered moderate when it is between 0.33 and 0.67, and considered outstanding when it is greater than 0.67 [54]. Every one of the components has a very modest impact on the whole. The values for CO, PI, PU, SA, ST and TP are about 0.463, 0.377, 0.310, 0.448, 0.267, and 0.243, respectively. Second, the term "standardized root" refers to the square residual (SRMR), and for the data to be considered normalized, its value must be less than or equal to 0.080 ([55–57]. The final number of 0.058 that the computation produces is satisfactory as a consequence of this. A model’s goodness of fit may be evaluated using a metric known as goodness of fit (GoF). According to the information shown in (1), it can be ranked as being either low (less than 0.10), small (between 0.10 and 0.25), moderate (0.25–0.36), or high (more than 0.36). Using the same measurement technique as [76–78], the result will determine whether to use it and datasets used in data analysis [79]. As a consequence, the GoF is at a high level, which is 0.501. Using the Eq (1) that GoF is presented below,

$$\text{GoF}=\sqrt{\bar{R^{2}}\times \bar{AvE}}=\sqrt{0.352\;x\;0.714}=\sqrt{0.251}=0.501$$ (1)

## Discussion

In this part, we will discuss how a suggested research model relates to previous research and how the results of these comparisons may impact theories and practices.

### Analyzed results

The findings of the PLS algorithm demonstrate indisputably that the three new variables included in the research, stakeholders (ST), transparency (TR), and social media network (SO), have a substantial correlation with perceived usefulness (PU) and confirmation (CO), and that they promote these two variables, satisfaction (SA) and participation intention (PI), when they are examined. In the second session, the hypothesis accepted all of the results and interpreted all of the correlations as true. The connection between the findings of the PLS algorithm is as follows: Social media networks that have grown into technology-based platforms, such as Facebook, Instagram, and WhatsApp, stimulate participation in social groups, which fosters transparency and allows communication among all stakeholders. As is evident from the current situation, complaints about any product or service acquired by people in the public or private sector will be used to post text or video clips on social media, with the opportunity to identify the entity principally responsible for that product or service. Therefore, this gives the perception of quicker access to problem solving than in the past, when people were obliged to compose a letter of complaint while juggling many obligations. Second, communities now have access to a valuable tool in the form of a social media network that allows for greater transparency (TP). This would increase any organization’s internal confirmation (CO) and perceived usefulness (PU). Lastly, the community’s desire to engage rises as a consequence of confirmation (CO) and perceived usefulness (PU). This makes the community want to participate more, which encourages more people to get involved in all communities and organizations.

### Theoretical implications

This research study broadens the scope of the inquiry and explains the links between multiple input factors by using theories to anticipate the outcomes, resulting in significant discoveries and new information for future research projects. The purpose of this study was to improve the original Technology Acceptance Model (TAM) by using an argument from the Expectation Confirmation Theory (ECT). Also, three new factors in this research, stakeholders (ST), transparency (TP), and social media network (SO), are substantially correlated with and promote confirmation (CO) and perceived usefulness (PU), followed by positive support for satisfaction (SA) and increasing participation intention (PI). This leads to the creation of a fresh conceptual framework for understanding user engagement in online communities. Second, this study contributes to our understanding of the conceptual model for managing online participation in any community by explicating and proving its relationship using widely recognized and sophisticated statistical methods. This study reviewed and maintained the original Technology Acceptance Model (TAM) to reflect the current context by including key factors such as stakeholders (ST), transparency (TP), and social networks (SO). Lastly, this article describes the experiments that will be carried out to support the assertions and highlights the significance of the research in understanding user participation in online communities.

### Practical implications

This study provides online behavior and practical insights on the participation of users in the online community through participatory management, which combines management by enabling people within the organization or those involved in the decision-making process to participate. Joint management requires management to demonstrate ingenuity and competence to achieve goals or overcome the myriad challenges that occur. This provides several advantages for the public and commercial sectors. Also, this research paper begins by introducing online community participation, which may be applied to efficiently capture and transmit pertinent information for community engagement and collaboration. Because social media can quickly and thoroughly share information on flash news and big events, researchers think this may increase efficiency. It is possible to govern departments in a manner that is both efficient and open to all parties’ examination. Each organization can interact more efficiently if they consider using social networks online. In this way, everyone can offer ideas and rely on the inventiveness and operational knowledge of others to help the agency achieve its objectives and address problems within its existing organization or domain. Second, this research provides models for understanding user behavior and participation in online communities that may function as mobile conference rooms for multi-team cooperation. Users may establish groups on online social media platforms such as WhatsApp or Facebook to discuss the event and chat, phone, video call, and live stream inside the group. Therefore, it is an ideal place or medium for gatherings, dialogues, voices, and coordination. Because it is possible to attend meetings regardless of location, distance is not an obstacle for meetings that will spend less on meeting preparations. Lastly, this conceptual model extends to participation in the online community as a means of building new businesses in a community, since social media is a relatively new industry that may be used to grow commerce and marketing. Online marketing, sometimes known informally as "online marketing," is rising in popularity due to the pervasiveness of social media in people’s lives today.

## Conclusions

This research uses a PLS structural equation model to analyze field data, including 627 consumers of community water from 14 communities. Consistent This study aims to extend the original Technology Acceptance Model (TAM) by using an argument from the Expectation Confirmation Theory (ECT) and three new variables derived from a synthesis of pertinent literature reviews: stakeholders, transparency, and social networks. The findings imply that social networks are a factor that supports stakeholders’ participation in the transparency process, which leads to satisfaction in virtual communities that share ideas with one another and achieve the goal of using participation. Furthermore, this research also adjusted and maintained the original expectation confirmation theory (ECT) and Technology Acceptance Model (TAM) to represent the dynamic character of the modern corporate environment by including key aspects such as stakeholders, transparency, and social networks.

In addition, this research also details the experiments that will be undertaken to confirm the hypotheses and illustrates the impact of understanding users’ participation in social networks on online participation and user behavior engagement across a range of social networks. In addition, the objective of this research is to establish a body of knowledge via public engagement in the construction of e-services and to support public participation (e-participation) by changing the organization’s position as a fully integrated digital facilitator. According to scholarly opinion, every organization must promote the expansion of easy and rapidly deployed social networks. Civil society must participate in e-services because it anticipates the organization to improve service quality while preserving resources. Furthermore, this conceptual paradigm has helped both the public and private sectors. For instance, it will assist any organization in establishing e-services or social networks as a "hub" between people and organizational entities. Therefore, in terms of the big organizations’ expectations for e-services, which should be more accessible and faster, Consequently, this research develops models and conceptual frameworks that encourage the development of efficient e-services, thereby facilitating the linkage of digital services to e-services, thereby reducing any paperwork process and enhancing access to e-services.

Furthermore, this study proposes a conceptual model that can be utilized as a guide for developing a social network-driven organization that drives work while allowing individuals to engage and measure progress. This is because when each stage can be validated, organization or agency activity will result in improved services. Furthermore, the conceptual model outlined in this document will help the organization develop a comprehensive plan for digital services that provides users with easy access to support information and services that are relevant to their jobs and daily lives, can be accessed from anywhere, and emphasizes the equality of all genders, ages, and educational levels participating in the social network system used in public administration to improve efficiency and customer satisfaction. In addition, e-services is a modern kind of digital administration that uses computer technology and communication networks to improve the effectiveness of operations and the quality of services provided to people. Moreover, e-services aim to provide on-line services and turn the organization into a customer-centric organization. The organization has made services accessible and convenient for the population. The organization is managed successfully with the aid of information technology. New technologies are altering the work practices of organizational personnel, and e-services may not be the only option in the future.

However, the organization must perform this task. All stakeholders, that is, your customers, initially anticipated that e-services would be provided through standardized channels, which corresponds to the findings of this article, and this expectation hastened the expansion of organization services through social networks. Using information in e-service systems to improve the efficiency of policy execution provides strategic advantages. Both the public and private sectors should be involved in making smart policy choices.

Finally, this study presents how social networks can improve water user participation in the community and then increase the intention of online community participation and influence the satisfaction of e-services. Developing a completely and effectively digital organization service system necessitates examining the design of a social network system in this context, in addition to linking everything via a digital system that is only concerned with the budget. All groups have equitable and complete access to different digitally or online-delivered organization services. In circumstances when users seek to submit certificates or papers proving qualifications from relevant authorities, a central data connection is developed by creating and altering the e-service process to be totally in the form of a social network. Future studies will focus on establishing a conceptual framework for future public administration. From a number of supporting variables, it is possible to identify public sector management that combines agile private management with the idea of excellence. The most important things are for the organization and any public company to use current technology to improve the role of the private sector in managing public services and to focus on providing services to people while keeping in mind the quality of life, the environment, and society.

### Limitations and further directions

This research explores the primary elements that influence the usage of the social network system, commonly known as e-service. Most of this study was undertaken remotely by volunteers representing each village during the COVID-19 epidemic. Therefore, researchers are unable to independently examine the study location in order to engage in the complete research process. Future studies may include three-stage follow-up surveys to demonstrate a causal association between factors. This research uses a simple random sample technique and focuses primarily on places where many individuals have experience with the provision of community water. Therefore, further research may increase the scope of future studies by collecting samples from other places or by surveying a greater number of urban water consumers, resulting in more convincing study findings. Additionally, the research area for future studies should be as broad as possible, which can be achieved by conducting more qualitative research in various study zones and in-depth interviews. In addition, the statistics and methodology employed in this investigation were chosen based on the aims of the study and results from relevant literature studies. To turn this study into a useful framework, you need to develop a conceptual framework and test and improve it with the right tools.

## Supporting information

S1 File Questionnaire. (PDF)
