# Supplementary material for: Understanding online behavior towards community water user participation: A perspective of a developing country
Source: PLoS One. 2022 Jul 28;17(7):e0270137. doi: 10.1371/journal.pone.0270137 (PMC9333291; doi:10.1371/journal.pone.0270137)
Supplement: S1 File — (PDF) [file pone.0270137.s001.pdf]

Questionnaire: Understanding online behavior towards community water user participation

- If you agree to participate in the research
  - you will be asked to complete a questionnaire. The questionnaire is comprised of 21 questions divided into three sections and should take approximately 15 minutes to complete.
  - The researcher does not request or store participants' personal information.
  - If new information about the research project becomes available, the researcher will inform the research participants as soon as possible and will not conceal it.
  - The risks associated with research participation You may feel uneasy or perhaps a little uneasy with certain questions. You have the option of not responding to those questions. You may terminate your participation in this project at any time. You will not be harmed by declining to participate in the research or withdrawing from the project without first giving it some thought. You will not be compensated for your time. And it is completely free.
  - The study enrolled a total of 1,000 individuals.
  - The total research period is 1 year (January 2021–December 2021).
  - Data collection period: 6 months after writing the report and the conclusion of this study.
  - The researcher will permanently destroy the data sheet and all related data. After completing the research (December 2021).

Part 1: General information of the respondents

Explanation: Please put a tick in (✓) that matches your actual statement.

1. Gender

( ) Male ( ) Female

2. Age

( ) Less than 30 years old ( ) 30 - 40 years old

( ) 41 - 50 years old ( ) More than 50 years old

3. Education level

( ) under bachelor's degree ( ) bachelor's degree ( ) higher than bachelor's degree

4. Community water usages (years)

( ) Less than 5 years ( ) 5-10 years

( ) 11-15 years ( ) More than15 years

Part 2: Factors of participation intention of E-services

Explanation: Please put a tick in (✓) that matches your actual statement.

**Subject**	**Level**				
	Least (1)	little (2)	Medium (3)	very (4)	the most (5)
**Perceived Usefulness**					
You think that social networks help you do your job faster and make it easier to find the information you need.					
You think that social networks help you to complete activities more rapidly.					
You believe that social network is beneficial and can help you perform better at work.					

Subject	Level				
	Least (1)	little (2)	Medium (3)	very (4)	the most (5)
**Satisfaction**					
You are satisfied with your social network experience.					
you satisfied with your capacity to utilize social network features					
You think you made the right decision to use social networks.					
**Perceived Usefulness**					
You think that e-services help you do your job faster and make it easier to find the information you need					
You believe that e-services assist you in completing activities more rapidly.					
You believe that e-services are beneficial and can help you perform better at work.					
**Confirmation**					
You are perpetually willing to help with community events.					
You take pride in your community membership.					
You believe it is critical to establish and maintain positive relationships with community-based organizations.					
**Social networks**					
You are always on social networks.					
You use social media to stay informed and connect with loved ones.					
You think that social media makes life easier.					
**Stakeholders**					
You think bringing people together for activities helps the community's image.					
You think that local and community activities help the community to work together and improve its economic well-being.					
You think it's vital to share responsibility for community and environment.					
**Transparency**					
The community should know and understand the rules before they are implemented, you believe.					
You think everyone should consider public topics like income and expenses.					
You think the organization office should share accurate data in an easy-to-read format.					

Subject	Level				
	Least (1)	little (2)	Medium (3)	very (4)	the most (5)
**Participation intention**					
You intend to continue utilizing social network rather than personally visiting an organization office.					
You plan to continue utilizing social network rather than discontinue doing so.					
You want to continue to use social network in the future					

Part 3: Additional Suggestions

……
……
……
……
……
……
……
……
……
……
……
……
……
……
………………………………………………
